# Oral vitamin A supplementation for ROP prevention in VLBW preterm infants

**DOI:** 10.1186/s13052-020-00837-0

**Published:** 2020-06-03

**Authors:** Francesca Garofoli, Donatella Barillà, Micol Angelini, Iolanda Mazzucchelli, Annalisa De Silvestri, Rosanna Guagliano, Lidia Decembrino, Chryssoula Tzialla

**Affiliations:** 1 Neonatal Unit and Neonatal Intensive Care Unit, Fondazione IRCCS Policlinico San Matteo, Pavia, Italy; 2 Eye Clinic, Fondazione IRCCS Policlinico San Matteo, Piazzale Golgi 19, 27100 Pavia, Italy; 3 Department of Internal Medicine and Therapeutics, Unit of Rheumatology, Università di Pavia, and Fondazione IRCCS Policlinico San Matteo, Pavia, Italy; 4 Clinical Epidemiology and Biometric Unit, Fondazione IRCCS Policlinico San Matteo, Pavia, Italy; 5 Pediatric Unit and Neonatal Unit. Ospedale Civile di Vigevano, ASST di Pavia, Vigevano (PV), Italy

**Keywords:** Retinopathy of prematurity (ROP), Vitamin A, Very low birth weight (VLBW) infant

## Abstract

Vitamin A administration may decrease any stage of retinopathy of prematurity (ROP) in preterm infants. To evaluate whether vitamin A oral supplementation could be preventive in ROP incidence and severity in VLBW infants, we compared results from 31 preterm infants, (< 1500 g or < 32 weeks) who, during a previous investigation, prospectively received 3000 UI/kg/die oral retinol palmitate drops, for 28 days, with 31 matching preterm newborns hospitalized in our NICU the same period, as control group. Although ROP incidence was similar, in the supplemented group, we had 9 cases of ROP grade 1, no ROP grade ≥ 2, in the un-supplemented group, 4 cases of ROP grade 1 and 6 ROP grade ≥ 2 (*p* = 0.018). The percentage of babies requiring treatment for ROP was 0 in treated and 16.6 in the un-treated group (*p* = 0.020). Moreover, Vitamin A administration showed a protective effect with an 88% risk reduction of developing severe ROP. Since vitamin A parenteral/IM administration presents some awareness, the results of this investigation may be important to plan further trials to confirm the usefulness of oral administration in mitigating the ROP severity of VLBW infants.

ClinicalTrials.gov NCT02102711; may 03/06/2014.

## Introduction

The results of a meta-analysis considering 67 studies and 21.819 infants, with the target to investigate the incidence and the possible prevention of retinopathy of prematurity (ROP) suggested that vitamin A supplementation and early, aggressive parenteral nutrition may reduce any stage ROP in preterm infants, but data relevant to vitamin A were from observational studies only [1].

ROP is a vasoproliferative vitreoretinal disorder arising from incomplete or immature retinal vascularization in preterm infants. It is a major cause of visual impairment or blindness in preterm infants and childhood blindness worldwide and [2]. ROP is a multifactorial disease with numerous risk factors: oxygen-therapy, low gestational age (GA), low birth weight (LBW), late onset sepsis (LOS), intraventricular haemorrhage (IVH), blood transfusion, and use of indomethacin, surfactant and erythropoietin. LBW or small gestational age (SGA) infants and these who require extended oxygen inhalation treatment have an increased incidence of ROP [3]. The International Classification of ROP (ICROP), revised in 2005 [4], classifies ROP in five stages of increasing gravity; the higher the stage of ROP, the more severe the disease is. Presence or absence of plus disease (dilation and tortuosity of the posterior retinal blood vessels) leads management and treatment decisions. Although the disease in its milder forms tends to regress spontaneously, in more severe cases and when not promptly and adequately treated, it can lead to unfavourable outcomes (retinal detachment and blindness). Retinal laser photocoagulation is the standard of care for treatment of ROP. However, it results a retinal damage and the loss of vision in the photocoagulation area and is not suitable for the more severe cases. Anti-vascular endothelial growth factor (Anti-VEGF) therapy has been a clinical option in second phase ROP treatment with neovascularization in babies with severe and aggressive ROP [5].

Preventive therapy for ROP is still lacking. In literature insulin-like growth factor-I, erythropoietin, propranolol, caffeine, antioxidants, and omega 3 poly-unsaturated fatty acids, Vitamin A supplementation have been suggested to have a preventative effect on ROP [6].

Vascular endothelial growth factor (VEGF) demonstrated to have a role in the pathogenesis of ROP, while vitamin A (retinoic acid) may prevent neovascularization resulting from oxygen-induced retinopathy, by downregulating VEGF expression thus deterring ROP onset [2]. The aim of this study was to evaluate whether vitamin A supplementation could be preventive in ROP incidence and severity in VLBW infants.

## Methods

We previously [7] performed a prospective clinical trial to evaluate vitamin A nutritional status in very low birth weight (VLBW) preterm infants. To this purpose, 31 consecutive preterm VLBW infants (< 1500 g or < 32 weeks, able to receive minimal enteral feeding, as inclusion criteria) were treated, for 28 days, with oral vitamin A (3000 UI/kg/die, retinol palmitate drops), in addition to the standard vitamin A amount by ESPGHAN guidelines, calculated on infant’s body weight [8]. Subsequent this part, in order to focus our attention to vitamin A preventive role in ROP incidence and severity, we retrospectively matched 31 VLBW preterm newborns (same inclusion criteria, hospitalized in our NICU the same period, with similar anthropometric and clinical characteristics, but not receiving vitamin A oral supplementation). Thus, we could have a balanced control group of 31 babies in order to evaluate ROP incidence and severity.

The parents of all the neonates signed the consent form for the anonymous use of clinical data for scientific purposes, approved by the Ethical Committee. ClinicalTrials.gov NCT02102711.

### Statistics

To summarize quantitative variable, mean and standard deviation (SD) were used, otherwise if not normally distributed, were described by median and Interquartile Range (IQR, 25th–75th percentile). Qualitative measures were compared using chi square test or Fisher exact test, as appropriate. Quantitative variables were compared using Student t-test for independent samples or Mann-Whitney test.

An ordinal logistic regression model was fitted using ROP (3 level variable no ROP vs ROP grade 1 vs ROP grade ≥ 2) as dependent variables and oxygen therapy, birthweight (BW), gender and vitamin A supplementation as independent factors. Results were expressed as Odds Ratio (OR) along with the corresponding 95% confidence intervals (CIs).

A *p*-value < 0.05 was considered statistically significant. STATA statistical package was used (release 16.0, 2019, Stata Corporation, College Station, Texas, USA).

## Results

Table 1 describes demographic and clinical characteristics of the 2 groups. No statistical difference was found in the descriptive variables. In particular, oxygen supplementation and incidence of comorbidities were similar in the 2 groups. Although ROP overall incidence was analogous in the 2 groups, in the supplemented group, we had 9 cases of ROP grade 1, no ROP grade ≥ 2, in the un-supplemented group, we accounted 4 cases of ROP grade 1 and 6 ROP grade ≥ 2 (*p* = 0.018). Among these 6 infants, 1 spontaneously recovered, 5 required treatment. Two received retinal laser photocoagulation therapy. Of the remaining 3 infants, who suffered from particularly severe and aggressive ROP, 1 was treated by anti-VEGF drugs, the other 2 infants, after anti-VEGF therapy, needed laser treatment too (Table 1). The percentage of preterm babies requiring treatment for ROP was 0 in treated and 16.6 in the un-treated group (*p* = 0.020). Moreover, at the multivariate analysis, Vitamin A administration showed a protective effect with an 88% risk reduction of developing severe ROP, independently from body weight at birth (BW), oxygen therapy and gender. Longer oxygen therapy was an independent risk factor while higher BW was a protective one (Table 2).

**Table 1:** Demographic and clinical data description

	Vitamin A Supplemented infants *N* = 31	Control infants *N* = 31	P
Maternal age, years, mean (SD)	33 (7)	32 (4.8)	0.6
Gestational age, weeks, mean (SD)	29 (2.33)	29 (2.66)	0.6
Anthropometric data at birth,			
Weight (g), mean (SD)	1134 (327)	1097 (234)	0.6
Length, (cm) mean (SD)	36.5 (3.4)	36.4 (2.8)	0.9
Head circumference, (cm) mean (SD)	25.8 (2.5)	25.9 (2.3)	0.8
SGA n (%)	7 (22.5)	7 (22.5)	1
IUGR n (%)	4 (12.9)	7 (22.5)	0.3
Male n (%)	16 (51.6)	19 (62.5)	0.4
Female n (%)	15 (48.4)	12 (37.5)	
Mode of delivery, n (%)			
vaginal delivery	5 (16)	9 (28.1)	0.2
caesarean section	26 (84)	22 (71.9)	
Apgar score at 1, mean (SD)	5.1 (2.4)	5.5 (2.1)	0.5
Apgar score at 5, mean (SD)	7.2 (2.2)	7.5 (1.6)	0.6
Stay in NICU, days, median (IQR)	57 (44–107)	69 (45–100)	0.6
Oxygen therapy, days, median (IQR)	26 (10–56)	27 (12–56)	0.7
Mechanical ventilation, days, median (IQR)	3 (1–7)	7 (1–17)	0.4
CPAP, days, median (IQR)	9.5 (4–33)	12.5 (6–41)	0.6
BPD n (%)	13 (41.9)	17 (54.9)	0.4
NEC n (%)	0 (0)	1 (3.3)	0.3
LOS n (%)	1 (3.2)	1 (3.2)	1
IVH grade ≥ 2 n (%)	0	4 (12.9)	0.1
ROP n (%)			
No ROP	22 (71.0)	21 (67.7)	
ROP grade 1	9 (29.0)	4 (12.9)	0.018
ROP grade ≥ 2 (with plus disease)	0	6 (19.4)	
ROP grade ≥ 2 n (%)			
No treatment		1 (16.7)	
Laser		2 (33.3)	
Anti-VEGF		1 (16.7)	
Anti-VEGF and laser		2 (33.3)	

*SD* standard deviation, *IQR* interquartile range, *SGA* small for gestational age, *IUGR* intrauterine growth restriction, *NICU* neonatal intensive care unit, *CPAP* continuous positive airway pressure, *BPD* bronchopulmonary dysplasia, *NEC* necrotizing enterocolitis, *LOS* late-onset sepsis, *IVH* intraventricular haemorrhage, *ROP* retinopathy of prematurity, *VEGF* vascular endothelial growth factor

**Table 2:** Risk factors in ROP

ROP	OR	95% CI	p
Oxygen (per day)	1.03	1.01–1.06	0.015
Birthweight (per gram)	0.99	0.98–0.99	< 0.001
Vitamin A controls/treated	0.12	0.02–0.69	0.018
Sex (M/F)	0.98	0.21–4.56	0.98

*OR* Odds ratio, *CI* confidence interval

## Discussion

The present study aimed to evaluate prevention ability of Vitamin A supplementation in ROP incidence and severity. Supplemented and control groups resulted appropriately matched and no significant differences were highlighted in the demographic and clinical characteristics (Table 1). Available studies suggest that parenteral/IM administration Vitamin A supplementation is able to prevent the complications of prematurity, at different dosage and regimen: 2000–3000 IU/kg/ day, 5000 IU 3 times/week and others [9]. Mactier et al. [10] demonstrated that early high-dose intramuscular vitamin A supplementation (10,000 UI/ 3 times weekly) for infants at risk of ROP improved retinal function at 36 weeks’ PMA, indicating that postnatal retinal development in preterm infants is at least partially dependent on an adequate supply of vitamin A. We previously demonstrated that oral formulation produces adequate plasma levels and is well tolerated; no adverse events attributable to Vitamin A supplementation have been observed [7].

Thus, the comparison between Vitamin A supplemented and un-supplemented VLBW infants showed that ROP incidence was higher in the control group and statistically different when considering ROP grade ≥ 2. In the un-supplemented group, retinopathy has evolved in the most severe stages, definitely requiring therapy, either laser photocoagulation, or anti-VEGF drugs or the 2 combined treatments. (Table 1) Moreover, an important and protective property was demonstrated by the odds ratio results relevant to vitamin A supplementation and ROP risk, confirmed by a risk reduction of  0.88. The study confirms the long-known role of oxygen therapy increasing the risk of ROP of 1.03 (Table 2).

Albeit study limitations (as sample size, retrospective comparison, not detailed anamnesis of parenteral /enteral nutrition), we believe that the investigation owns interesting results. Since vitamin A parenteral/intramuscular administration presents some awareness, (photodegradation and vitamin adsorption to the plastic of the intravenous administration set/ discomfort and trauma associated with injection) and the preventive use of retinol supplementation is still a matter of debate, the outcomes of this investigation may be important to plan future trials to confirm the usefulness of oral administration in mitigating the ROP severity of VLBW preterm infants.

## Data Availability

The datasets used and/or analyzed during the current study are available from the corresponding author on reasonable request.
